# Particular shape of the tongue and benign migratory glossitis

**DOI:** 10.11604/pamj.2018.30.11.15490

**Published:** 2018-05-07

**Authors:** Cinzia Casu, Luca Viganò

**Affiliations:** 1 Private Dental Practice, Cagliari, Italy; 2 University of Milan, Department of Radiology, Milano, Italy

**Keywords:** Benign migratory glossitis, bruxism, tongue shape

## Image in medicine

A 33-year-old male patient came to our observation for a slight discomfort in the left side of the tongue. The patient reported good general health and that he have sometimes taken benzodiazepine in periods of stress. The dental abrasion showed the presence of bruxism. On an objective examination we could notice the simultaneous presence of a "toothed tongue" and a slightly area of erosion on the left side of the dorsal tongue, with an irregular white halo around it. This lesion, appeared from some days, is symptomatic. The diagnosis is compatible for benign migratory glossitis. The patient claimed to have never suffered of skin diseases strongly related to migratory glossitis. It is possible that the bruxism may have determined the presence of that lateral tongue shape and that stress may be the main cause of the appearance of migratory glossitis. The simultaneous presence of the two conditions has never been documented in the scientific literature and we do not exclude that there may be a correlation to the genesis of both lesions. Sometimes the presence of a tongue with this morphology could be the result of a trauma or a surgery. The lingual erosion can be placed in differential diagnosis with a heat burn or a chemical burn.

**Figure 1:**
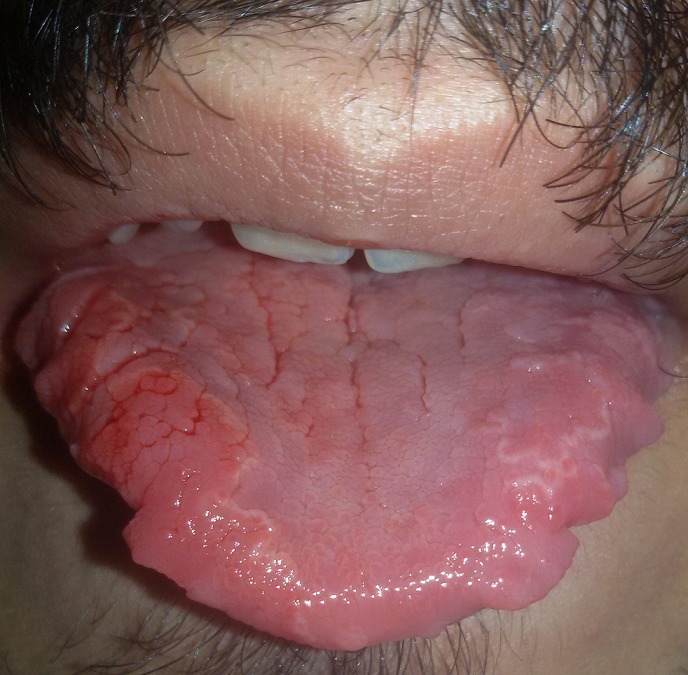
"toothed tongue" and benign migratory glossitis

